# Assessment of Fluoride Content in Water and Its Impact on the Intelligence Quotient of School Children Aged 12–13 Years

**DOI:** 10.7759/cureus.30157

**Published:** 2022-10-10

**Authors:** Diljot Kaur, Kanwalpreet Kaur, Ashish Sharma, Hemant Goyal, Akshay Pahuja, Dinesh Solanki

**Affiliations:** 1 Public Health Dentistry, Genesis Institute of Dental Sciences and Research, Ferozepur, IND; 2 Pediatric and Preventive Dentistry, The Tooth Clinic, Ludhiana, IND; 3 Neurology, All India Institute of Medical Sciences Bilaspur, Bilaspur, IND; 4 Prosthodontics and Implantology, Maharana Pratap College of Dentistry, Gwalior, IND; 5 General Medicine, Genesis Institute of Dental Sciences and Research, Ferozepur, IND; 6 Pediatric and Preventive Dentistry, Vyas Dental College & Hospital, Jodhpur, IND

**Keywords:** iq and flouride content in water, rajasthan, jaipur, schoolchildren, iq, water, fluoride

## Abstract

Background: The preliminary study was undertaken with the aim to assess the effect of fluoride content in water on the Intelligence Quotient (IQ) of school children aged 12-13 years residing in areas that differ with respect to fluoride levels.

Materials and Methods: The IQ was measured using Raven’s Colored Progressive Matrices in 90 children, who were life-long residents in three villages (30 children each) of similar population size but differing in the level of fluoride in drinking water. Urinary fluoride concentration was measured using the selective ion electrode technique. One-way ANOVA was used for the statistical analysis of the data.

Results*:* Children who lived in locations with fluoride levels of 1.60, 6.70, or 2.80 parts per million in their drinking water had urinary fluoride concentrations of 1.60, 6.82, or 2.69 parts per million, and IQ scores of 16.77 + 8.24, 19.36 + 9.98, or 21.87 + 7.47, respectively.

Conclusion: The results indicated that there was a positive correlation between excess fluoride in drinking water and IQ.

## Introduction

All around the world, endemic fluorosis has grown to frightening proportions. India is located in a fluoride belt. In 18 of the 32 Indian states, it is a serious public health issue. It has been shown that 177 or so districts are affected by high fluoride content in water. According to a United Nations Children's Fund (UNICEF) report, 20% of the fluoride-affected communities worldwide are in India, and 10% of those are in Rajasthan. The largest state in India, Rajasthan, has a surface area of 342,000 square kilometres and accounts for 10.41% of the country's land area, 5.5% of its population, but only 1% of its water resources [1,2]. In all 33 of Rajasthan's districts, the groundwater has high levels of fluoride. In many parts of Rajasthan, people are required to drink water that contains up to 44 mg/l of fluoride [3].

According to research, fluoride can cause negative biochemical and functional alterations in the developing human brain [4]. High levels of fluoride in drinking water may have a negative impact on intelligence, which could be a major health issue. A robust and continuous link between fluoride exposure and low intelligence quotient (IQ) was found in the meta-analyses by Lu et al., Sharma et al., and Tang et al. [5-7].

Due to its high fluoridation levels, Rajasthan state is a prime location for the emergence of IQ-related issues [5]. A preliminary cross-sectional study was done to evaluate and compare the impact of fluoride levels in water on the IQ of school children aged 12-13 years after a survey of the scientific literature revealed no similar study in this region.

## Materials and methods

A cross-sectional study was undertaken on 90 school children from September 2011 to October 2011 in Jaipur district, Rajasthan. Data regarding fluoride concentration in water and the list of government schools in the Jaipur district were taken from the Public Health Engineering Department and Shiksha Sankul, respectively. The plan was reviewed and approved by the Institutional Ethical Committee of Genesis Institute of Dental Sciences and Research, Ferozepur, Punjab, India (Ethical clearance number: JDC68/2010). School authorities and parents provided written informed consent prior to the conduct of the study. A pilot-tested questionnaire (= 0.85), completed with the assistance of parents, provided information on demographic characteristics, food, and oral hygiene practices. It was drafted in English and translated into a local dialect.

Using a stratified cluster random sampling, three villages, namely Dhand of Amer Tehsil, Mohanpura, and Muhana of Sanganer Tehsil with approximately similar population sizes but differing with respect to fluoride content in drinking water were selected. Iodized salt was used in all the areas. The total number of school children aged 12-13 years at Dhand, Mohanpura, and Muhana was 35, 42, and 39, respectively. Children with a history of trauma or injury to the head and those affected by any congenital or acquired neurological disorders or psychological disorders were excluded from the study. Thirty children were randomly allocated from each school into their respective groups. The children were divided into three groups: Group A (Fluoride concentration of 2 ppm), Group B (Fluoride concentration of 5 ppm), and Group C (Fluoride concentration of 2-5 ppm).

IQ testing procedures and standards

The IQ of the children was measured using Raven's Coloured Progressive Matrices™ intelligence test [8], which consists of a series of multiple-choice questions. Before administering the test, a friendly explanation of the important instructions was given by a single examiner to avoid mental stress for those taking the test. Children were made to sit in a manner to ensure that they couldn’t talk with each other. The details of intellectual ability ranking and interpretation are given in Tables 1, 2.

**Table 1:** Ranking of intellectual ability according to Raven's Coloured Progressive Matrices™

Score	Percentile
18	5^th^
22	10^th^
26	25^th^
31	50^th^
33	75^th^
34	90^th^
35	95^th^

**Table 2:** Interpretation of intellectual ability IQ: intelligence quotient

IQ Range	Interpretation
>5^th ^percentile	Intellectually Impaired
5^th^ - 25^th^ percentile	Below Average
25^th^ – 75^th ^percentile	Average
75^th^ – 90^th^ percentile	Above Average
95^th^ percentile	Intellectually Superior

Determination of the fluoride level in urine samples

Non­reactive 60 ml plastic containers with respective codes were handed over to children for urine samples. The collected samples were stored in an ice box and were submitted to the Rajasthan State Pollution Control Board within eight hours of the collection for the analysis of fluoride content. The concentration of fluoride in urine was assessed using the Selective Ion Electrode Technique [4,8-10]. In order to ensure reliability, the sample was appraised thrice, and the mean value was documented. One-way ANOVA test and paired t-test were used for statistical analysis of the data, and a p-value less than 0.05 was considered significant.

## Results

A highly statistically significant difference (p<0.001) was observed in urinary fluoride levels. Fluoride excretion in group B was 6.82, in Group C it was 2.69, and in Group A it was the least with values of 1.60. In comparison among the three groups, the highest fluoride excretion in urine was 6.82 ppm in Group B where the fluoride level in the drinking water was 6.70 ppm (Tables 3, 4).

**Table 3:** Fluoride excretion from urine in groups ppm: parts per million

Fluoride levels in water	Fluoride excretion from urine
Group-B (6.70 ppm)	6.82
Group-C (2.80 ppm)	2.69
Group-A (1.60 ppm)	1.60

**Table 4:** One-way ANOVA for fluoride excretion from urine HS: highly significant; SS: sum of squares; df: degree of freedom

Source of Variation	SS	df	F	p-value	Significance
Between Groups	454.775	2	324.30	< 0.001	HS
Within Groups	61.001	87			
Total	515.776	89			

When the groups were analyzed for both intra- and intergroup comparisons, the results of both showed that there was a statistically significant difference in the levels of urine fluoride following excretion. No statistically significant correlation (p> 0.05) existed between fluoride excretion and IQ in Group A children. But there was a statistically significant correlation between fluoride excretion and IQ level in Group B (p<0.01) and Group C (p< 0.05). As the level of fluoride ion concentration in urine increased, there was a significant decrease in IQ level (Table 5). This suggests that IQ has an impact on fluoride excretion through the body.

**Table 5:** Correlation between fluoride excretion from urine and IQ level of schoolchildren of all the groups IQ: intelligence quotient

	r-value	p-value	Significance
Group A	- 0.161	> 0.05	Not Significant
Group B	-0.485	< 0.01	Significant
Group C	-0.334	< 0.05	Significant

## Discussion

It is well known that the prevalence of fluorosis varies greatly depending on the number of fluoride ions present in the groundwater. Most of the fluoride in drinking water is due to leaching from geological formations. Fluoride toxicity, therefore, poses a threat to a significant portion of India. The goal of the current study was to determine how fluoride concentration in water affected the IQ of schoolchildren aged 12-13 years who attended schools in areas with varying fluoride levels. In contrast to urban regions, where water filtration devices are more readily available, the study focused on children who live in rural locations. Only children who were born and raised in the same region were chosen, taking all necessary precautions. Raymond Cattell asserts that intelligence increases until the age of 15 and thereafter remains constant. Additionally, examining these age groups made it easier to compare the results of this study with those of numerous other studies in which kids in a comparable age group were examined [4,5,9]. Children's IQs were measured using Raven's Coloured Progressive Matrices test, which is a "culturally fair" test suited for comparing children in terms of their immediate capacities for observation and clear reasoning. Despite the fact that this test was created to cover as many different types of mental abilities as possible, there are a few potential flaws that should be taken into account. The results show relative intelligence rather than pure intelligence. The term "intellect" is broad and encompasses qualities like inventiveness, tenacious curiosity, logical reasoning, problem-solving ability, critical thinking, and adaptability. These various facets of intellect are not interdependent. This test does not assess other aspects of intelligence; it only assesses observation, clarity of thought, and logical reasoning. Since additional IQ categories are not taken into account, an IQ test cannot provide a comprehensive picture of a person [7,8].

Fluoride intake increased when drinking water had a high fluoride content, and this was supported by higher urine fluoride levels. The urine fluoride level was examined using the selective ion electrode technique because the kidney is the main organ for the excretion of fluoride. This allowed researchers to determine the pace or degree of exposure to fluoride [4,8-10]. The results of the study showed that there was a significant negative correlation between intelligence and urine fluoride levels in groups B and C, but not in group A. These results were in line with those of a meta-analysis carried out by Tang et al. to determine whether fluoride exposure raises the likelihood of low IQ in China [7]. A continuous and significant correlation between fluoride exposure and low IQ was revealed in a qualitative evaluation of 16 case-control studies that evaluated the emergence of low IQ in kids who had been exposed to it earlier in life. The odds ratio of IQ in endemic fluoride locations in comparison to non-fluoride areas or places with only a minor amount of fluoride was also determined by the meta-analyses of the case-control studies. According to the summarised weighted mean difference, children who live in a fluorosis area have a five-fold higher risk of developing low IQ than children who live in a non-fluorosis area or a slight fluorosis area using a fixed-effect model and a random-effect model [6]. The study's findings concurred with a study by Trivedi et al. in which the IQ of 190 schoolchildren living in two villages of Gujarat, India, with comparable socioeconomic, dietary, and educational situations but varied fluoride levels in their drinking water was determined [4]. Children who lived in the high fluoride area had urine fluoride levels that were higher (6.13 mg/L) than children who lived in the low fluoride area (drinking water = 2.010 mg/L, urinary fluoride levels of 2.300 mg/L). A questionnaire developed by Professor JH Shah and standardized on the Gujarati community was used to determine the children's intelligence quotient (IQ), which had a 97% reliability rate in comparison to the Stanford-Binet Intelligence Scale. The mean IQ of the 101 children in the lower fluoride area (104.44) was substantially higher than that of the 89 children in the high fluoride area (91.721). Long-term exposure to high fluoride levels can affect how a person's mind grows [11-13]. IQ and urine fluoride levels showed a substantial unfavorable connection as well [9], and the findings of the study agreed with those of Poureslami et al. [13].

The results of the current investigation were similar with those of the study by Eswar et al., which was a cross-sectional study conducted to compare the IQ of schoolchildren aged 12-14 years living in a high fluoride village with those of a comparable group of children in a low fluoride village [9]. A total of 65 students from the low fluoride village (0.29 ppm) and 68 students from the high fluoride village (2.45 ppm) were compared. The fluoride ion selective electrode method was used to evaluate water fluoride levels. Raven's Standard Progressive Matrices exam [8] was used to gauge IQ levels. Two Z tests and a chi-square test was employed to statistically analyze the data. In comparison to the low fluoride village, where the mean IQ score was 88.81, it was determined that the children in the high fluoride village had a lower mean IQ score (86.31). The difference, however, was not statistically significant (p = 0.30). Children with IQ scores below 90 were found to be 43/68 (63.2%) in the high fluoride area and 31/65 (47.7%) in the low fluoride area. This difference was almost statistically significant (p = 0.006). The study's general conclusion was that high fluoride water was associated with lower IQ, although no correlation between the fluoride level in the drinking water and the IQ scores of the children was shown to be statistically significant [9].

There is no obvious explanation for how fluoride lowers IQ. Fluoride's capacity to pass through the blood-brain barrier and cause biochemical and functional impairment of the nervous system throughout prenatal and developmental stages of infancy and childhood is one of the main causes of lower intellect in children exposed to high amounts of fluoride. Additionally, cholinesterase activity, which is responsible for hydrolyzing choline esters, is known to be negatively impacted by fluoride. The transmission of nerve impulses in brain tissue could be affected by this toxic effect's altered acetylcholine usage. Changes in membrane lipids may be the origin of this condition, according to a paper by Guan et al. [14], which found changed levels of phospholipids and ubiquinone in the brains of rats with chronic fluorosis. It is also well known that fluoride can cross the placenta and reach the fetus. If the child is exposed to fluoride continuously throughout childhood, it may negatively affect the growing brain and lower the child's IQ. However, it is well established that a variety of factors, such as inheritance, genetic flaws, and environmental circumstances, can affect IQ. Nutrition, prenatal care, breastfeeding, endemic iodine shortages, and parental IQ are examples of factors. IQ development is significantly influenced by physical abuse, academic achievement, and maternal fluoride exposure throughout pregnancy. Thus, one of the many environmental influences affecting children's IQ is fluoride in drinking water. To confirm the results of the current study, researchers need to conduct more longitudinal studies with larger sample sizes and take into account different confounding factors [8,9,14].

In a prior study, rats given sodium fluoride produced plasma levels of fluoride that were comparable to those in humans who drank water with 4 ppm (parts per million) of fluoride and had symptoms similar to those with attention deficit hyperactivity disorder [14]. Fluoride-related dental and skeletal issues in people have a long history of research. According to Guan et al., chronic fluorosis altered the amounts of phospholipid and ubiquinone in rat brains, and these alterations in membrane lipids may contribute to the pathophysiology of this condition [15-19].

According to Raven et al., fluoride seriously hinders the production of bone matrix, which leads to a decrease in bone calcification [18]. They noticed that increased fluoride levels cause serious health problems in over 20 countries. Additionally, it was found that increased fluoride levels in drinking water were linked to stillbirths, early infant death, and birth abnormalities. Increased fluoride content can also cause neurological problems. Fluoride is frequently marketed as a way to prevent dental cavities, but too much of it can have negative consequences. One of the things that affect intellectual development, according to the research, is being exposed to too much fluoride over time [8]. A cross-sectional study was conducted to determine the impact of long-term high fluoride exposure on children's IQ in Iran's Kerman province's urban environment [19]. Koohbanan City and Baft City, two metropolitan areas with comparable socioeconomic standing and cultural values but varying fluoride levels in their drinking water, were selected. Koohbanan City and Baft City had fluoride concentrations of 2.38 mg/L and 0.41 mg/L, respectively. The study involved 119 kids between the ages of six and nine years and Raven's Progressive Matrices exam was used to calculate the children's IQs. The children's respective mean IQ scores in the high fluoride location (Koohbanan) and low fluoride area (control group) were 97.80 and 91.37. The IQ scores of the two groups showed a statistically significant difference (p 0.05). It was determined that even though fluoride is strongly advocated for use in preventing dental caries, excessive fluoride consumption has negative effects on oral health and can result in dental fluorosis.

The limitations of the study include the small sample size and the fact that longitudinal effects were not estimated in the study. 

## Conclusions

According to the findings of the study, a positive correlation was found between excess fluoride in drinking water and IQ. Thus, comprehensive measures should be planned. The presence of fluoride in children's drinking water is just one of the many environmental factors that can have an effect on a child's intelligence level. Therefore, additional longitudinal studies using larger sample sizes and taking into account different confounding factors are required to confirm the findings of the current study.
